# Branched-Chain and Aromatic Amino Acids in Relation to Fat Mass and Fat-Free Mass Changes among Adolescents: A School-Based Intervention

**DOI:** 10.3390/metabo12070589

**Published:** 2022-06-24

**Authors:** Magnoudewa Priscille Pana, Pierre Ayotte, Elhadji Anassour-Laouan-Sidi, Edouard Suhas, Clémence Mahana Iti Gatti, Michel Lucas

**Affiliations:** 1 Population Health and Optimal Health Practices Research Unit, CHU de Québec-Université Laval, Québec City, QC G1V 4G2, Canada; mapan16@ulaval.ca (M.P.P.); pierre.ayotte@inspq.qc.ca (P.A.); elhadji-anassour.laouan-sidi@inspq.qc.ca (E.A.-L.-S.); 2 Department of Social and Preventive Medicine, Faculty of Medicine, Université Laval, Québec City, QC G1V 0A6, Canada; 3 Centre de Toxicologie du Québec, Institut National de Santé Publique du Québec, Québec City, QC G1V 5B3, Canada; 4 Institut National de Santé Publique, Québec City, QC G1V 5B3, Canada; 5 Non-Communicable Diseases Unit, Oceanian Islands Ecosystems, UMR 241, Louis Malardé Institute, Papeete 98714, French Polynesia; esuhas@ilm.pf; 6 Laboratory of Marine Biotoxins, Institut Louis Malardé (ILM), UMR 241-EIO (IFREMER, ILM, IRD, Université de Polynésie Française), Papeete 98713, French Polynesia; cgatti@ilm.pf

**Keywords:** branched-chain amino acids, aromatic amino acids, obesity, weight loss, fat mass, fat-free mass, insulin resistance, adolescents

## Abstract

Plasma levels of branched-chain amino acids (BCAA) and aromatic amino acids (AAA) are considered early metabolic markers of obesity and insulin resistance (IR). This study aimed to assess changes in plasma concentrations of BCAA/AAA and HOMA-IR2 (homeostasis model assessment of IR) after intervention-induced modifications in fat mass (FM) and fat-free mass (FFM) among French Polynesian adolescents. FM, FFM, plasma levels of BCAA and AAA, HOMA-IR2 were recorded at baseline and post intervention among 226 adolescents during a 5-month school-based intervention on diet and physical activity. Participants were divided into two subgroups according to their college attendance status which determined their intervention adherence: externs/half-residents (n = 157) and residents (n = 69). Four ordinal categories of body composition changes post-intervention were created for the analysis (FM^gain^/FFM^lost^ < FM^gain^/FFM^gain^ < FM^lost^/FFM^lost^ < FM^lost^/FFM^gain^). After 5 months, changes in BCAA (*p*^−trend^ < 0.001) and AAA (*p*^−trend^ = 0.007) concentrations were positively associated with ordinal categories of body composition. HOMA-IR2 significantly decreased with FM^lost^ (−0.40; 95% CI, −0.60 to −0.20) and increased with FM^gain^ (0.23; 95% CI, 0.11 to 0.36). Our results suggest that FM loss is associated with a decrease in concentrations of obesity and IR metabolic markers which is more substantial when FM loss is accompanied with FFM gain.

## 1. Introduction

Obesity in childhood and adolescence is a major public health issue in both developed and developing countries [1]. About 107 million children worldwide, under 20 years of age, were living with overweight or obesity in 2015, and in some countries, the number of children with overweight has doubled since 1980 [2]. French Polynesia has not been spared from the child obesity pandemic. In 2011, a study showed that 60% of Polynesian adolescents were living with overweight, of whom 28% were obese [3]. The nutritional transition that occurred over the past few decades has led to unhealthy diets which in conjunction with decreased physical activity might be responsible for the rise of obesity in this population [4]. These lifestyle changes fueled an epidemiological transition characterized by the decline of infectious diseases and the rise of obesity-related chronic diseases such as diabetes, hypertension, and cancer [5]. The younger generation of Polynesians seems particularly affected by this phenomenon. In 2009, the country had one of the highest prevalence rates of childhood obesity in the world, with 34% of obese children aged 5 to 14 years, compared to 20% in the United States [5]. However, only few investigations and obesity interventions focused on this region.

Childhood obesity and overweight-related complications are diverse; the first being its persistence into adulthood. In a systematic review of the consequences of childhood obesity, it is estimated that, compared to normal-weight children, overweight children are at least twice as likely to be overweight in adulthood. One study even reported a ten-fold increase in risk [6]. A recent study predicting obesity among future adults pointed out that 57% of today’s children will be obese by the time they reach 35 years old [7]. Other than the risk of adulthood obesity, adolescents with obesity or overweight are prone to obesity-related diseases during their adolescence [2,6,8]. A previous study (“La transition alimentaire et sanitaire en Polynésie française”) conducted among French Polynesian natives of the Austral Islands in 2007 revealed that a significant number of adolescents suffered from prediabetes and hypertension associated with a high rate of obesity [5,8,9]. As childhood obesity can profoundly affect children’s physical health, social, and emotional well-being, and self-esteem [6], prevention efforts are emerging as a public health priority.

School-based interventions are a common choice for obesity prevention because they are ideal for promoting healthy diets, healthy behaviors, and implementing healthy policies [10,11]. Several methods are used to evaluate the effects of such interventions on body changes, but no consensus has yet been reached [12]. Although changes in body mass index (BMI) and overall body weight can be used to estimate the impact of an obesity-related intervention, studies suggest that body fat percentage is a more physiologically significant measure [12]. Moreover, studies on weight loss programs have pointed out that body weight loss after these programs was not equal to fat loss, and that almost 25% of weight loss observed post-intervention is due to a reduction in lean tissue [13]. Those studies also showed that the concurrent analysis of body fat and lean tissue provides an improved interpretation of intervention success compared to inference derived from BMI alone. However, modifications in weight and body fat are not the only measurable effect of obesity-related interventions. In fact, these changes are more relevant health-wise when they affect the metabolic system.

Lately, a major challenge that arose in the obesity research area is the early detection of its related chronic diseases. The emerging field of metabolomics has led to the identification of potential biomarkers for type 2 diabetes (T2D) and insulin resistance (IR) [14]. Branched-chain amino acids (BCAA), aromatic amino acids (AAA), and acylcarnitines have been identified as early markers of obesity and IR that may improve the prevention of cardiometabolic diseases [15,16]. In addition, Gaggini et al., using direct measure of IR through in vivo tracers, reported that the concentration of amino acids was associated with obesity and IR, especially in subjects with obesity, likely due to increased IR and protein catabolism [17].

Few studies on obesity-related interventions in adolescents focused on metabolomic changes following weight reduction, especially changes in circulating BCAA/AAA concentrations. To date, no study has examined the changes in circulating levels of these metabolites in relation to specific body composition changes in adolescents characterized through concomitant analysis of fat mass (FM) and fat-free mass (FFM). For this study, we assessed data obtained from a school-based 5-month intervention carried out among French Polynesian adolescents called the “Ressources Alimentaires et Santé aux Australes (RASA)” study. We aimed to examine changes in BCAA, AAA, and IR in relation to FM and FFM changes post intervention. We hypothesized that BCAA and AAA concentrations will be associated with weight changes and body composition changes following the 5-month RASA physical activity and diet intervention in Polynesian adolescents. More specifically, a reduction in BCAA and AAA concentrations should be noted in adolescents who lost fat mass, while an increase in BCAA and AAA concentrations should be observed in those who gained fat mass.

## 2. Results

The average age of participants was 13.5 (1.6) years and 47.8% were girls (Table 1). Externs/half-residents were one-year younger than residents. Overall overweight/obesity prevalence was 58.9%, with 34.1% of adolescents exhibiting obesity. No statistically significant differences were noted for height, weight status, and FFM percentages between the two groups.

### 2.1. Overall Weight Changes

All results concerning anthropometric measures of adolescents before and after the intervention were previously reported [17]. Those results showed that 77 (34.1%) adolescents had lost weight post intervention and 149 (65.9%) had gained weight. After 5 months of follow-up, weight increased significantly among externs and half-residents, in contrast to the group of residents [18]. Compared to the residents, the adjusted difference in weight change for externs/half-residents was 2.3 kg (95% CI, 1.1 to 3.8) [18]. The proportion of adolescents who lost weight increased (*p* < 001) with exposure to food and physical activity commitments (residents being the most exposed): 27% of externs/half-residents and 58% of residents [18].

### 2.2. Changes in BCAA/AAA Concentrations and HOMA-IR2 According to Weight Status at Baseline

At baseline, adolescents with obesity had higher BCAA and AAA concentrations than those with normal weight and those with overweight (Table 2). After the 5-month intervention, we noted a mean decrease of −3.25 (95% CI, −6.43 to −0.07) in BCAA concentrations and −1.16 (95%CI, −2.16 to −0.15) in AAA concentrations for residents whereas a mean decrease of −4.53 (95% CI, −6.39 to −2.68) in BCAA concentrations and −2.44 (95% CI, −3.27 to −1.61) in AAA concentrations were observed for externs/half-residents (Table 2). According to stratification analysis by IOTF weight status at baseline, the time effect on metabolite changes was statistically significant among adolescents with overweight and obesity, but the group x time effect was only statistically significant among adolescents with obesity (Table 2). Analysis according to HOMA-IR2 tertiles at baseline revealed that adolescents with lower IR experienced a greater decrease in BCAA and AAA concentrations compared to those with a higher IR (Table S1).

**Figure 1:**
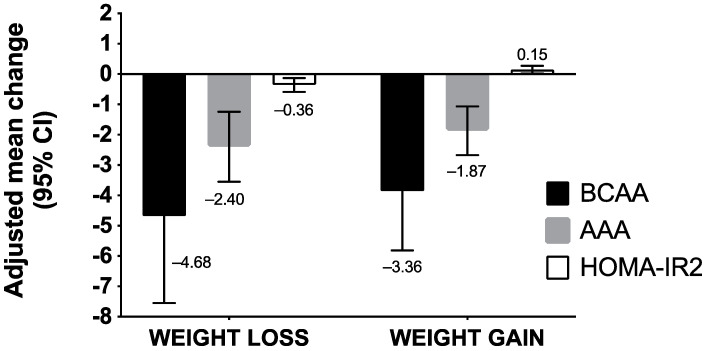
Adjusted mean differences of BCAA/AAA concentrations and HOMA-IR2 index according to intervention-induced weight changes in adolescents enrolled in the RASA study. Abbreviations: AAA, aromatic amino acids; BCAA, branched-chain amino acids, FM, fat mass; FFM, fat-free mass; HOMA-IR2, homeostasis model assessment of insulin resistance. We performed a multivariate analysis of variance using PROC GLM procedure (a method of least squares to fit general linear models). We assessed the changes in BCAA, AAA concentrations, and HOMA-IR2 index according to changes in weight after the intervention. Models were adjusted for baseline score of BCAA/AAA concentrations, sex, and age, intervention group, and IOTF weight status.

Figure 1 summarizes how BCAA/AAA levels and HOMA-IR2 index vary according to weight changes. Both adolescents who experienced weight loss or gained weight had significant changes in their BCAA/AAA concentrations and HOMA-IR2 index. Among adolescents who lost weight, BCAA concentrations decreased by −4.68 (95% CI, −7.55 to −1.81), AAA by −2.40 (95% CI, −3.55 to −1.24), and HOMA-IR2 by −0.36 (95% CI, −0.59 to −0.13) (Table S1). Among adolescents who gained weight, we noted a decrease of −3.86 (95% CI, −5.81 to −1.91) for BCAA and −1.87 (95% CI: −2.67 to −1.07) for AAA, whereas an increase of 0.15 (95% IC, 0.02 to 0.27) was observed for the HOMA-IR2 index (Table S2).

### 2.3. Changes in BCAA and AAA Concentrations and HOMA-IR2 According to Fat Mass and Fat-Free Mass Changes

**Figure 2:**
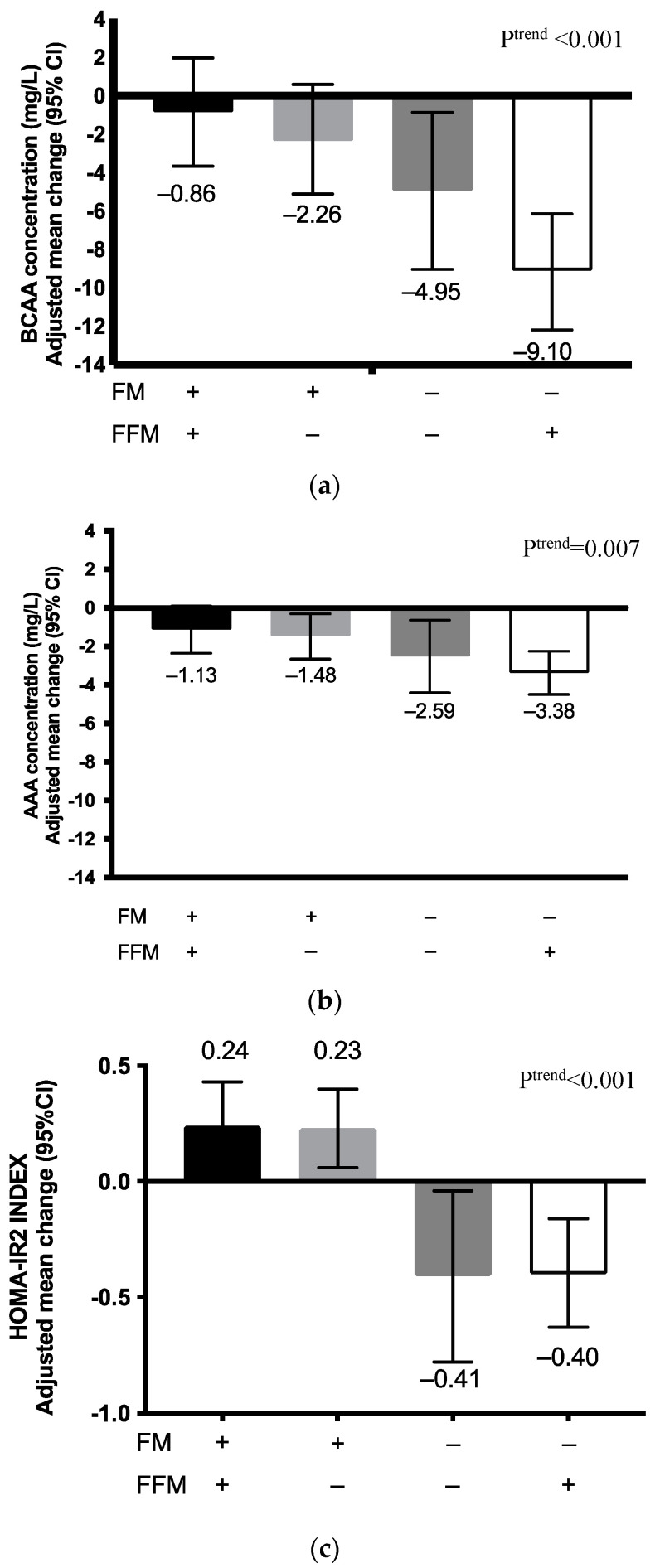
Adjusted mean differences in (**a**) plasma BCAA concentrations; (**b**) plasma AAA concentrations, and (**c**) HOMA-IR2 indices according to body composition change categories (FM and FFM) among adolescents participating in the RASA study. Abbreviations: AAA, aromatic amino acids; BCAA, branched-chain amino acids, FM, fat mass; FFM, fat-free mass; HOMA-IR2, homeostasis model assessment of insulin resistance, (+), gain; (−), loss. We used SAS software PROC GLM CONTRAST to assess changes in BCAA/AAA concentrations and HOMA-IR2 index according to the four body composition change categories created. Models were adjusted for baseline BCAA/AAA concentrations, sex, age, and weight status. We also tested for linear trends across body composition change categories using the same procedure.

Compared to the previous analysis focusing on weight status, analysis in terms of body composition changes yielded different results. Among adolescents who gained FM, changes in BCAA concentrations were not statistically significant, regardless of the changes in FFM: adjusted mean difference was −0.86 (95% CI: −3.68 to 1.97) for FM^gain^/FFM^loss^ and −2.26 (95% CI, −5.12 to 0.59) for FM^gain^/FFM ^gain^ (Figure 2, Table S3). In contrast, among adolescents who lost FM, we noted statistically significant decreases in BCAA levels in both FFM change subgroups: adjusted mean difference was −4.85 (95% CI: −9.04 to −0.87) for FM^loss^/FFM^loss^ and −9.10 (95% CI, −10.1 to −5.20) for FM^loss^/FFM^gain^. For AAA concentrations, the decrease of plasma levels was statistically significant for all groups except in the FM ^gain^/FFM^loss^ group (−0.86 (95% CI: −3.68 to 1.97)).

As for changes in HOMA-IR2 index, FM change was the major determinant. The index increased in participants who gained FM (0.23 (95% IC, 0.11 to 0.36)), but decreased in those who lost FM (−0.40 (95% IC, −0.60 to −0.20)). No statistically significant difference was noted for changes in HOMA-IR2 index when FM loss was combined with gain or loss of FFM.

## 3. Discussion

In the present study, we used data from a school-based intervention on obesity among Polynesian adolescents to assess changes in early metabolic markers of obesity and IR. The primary assumption of the interventional studies is that weight loss resulting from such interventions induces metabolic changes in the subjects. We therefore measured changes in levels of BCAA/AAA and HOMA-IR2 index in relation to body composition changes post intervention (fat mass and fat-free mass).

Overall, our results indicate that the 5-month intervention on diet and physical activity resulted in lower plasma levels of BCAA and AAA for most adolescents. We had assumed that the residents’ group would experience a greater decline in metabolite concentrations compared to the half-residents/externs. However, contrary to our assumptions, the half-residents/externs experienced greater decreases in concentrations for BCAA and AAA, with some overlapping confidence intervals. Comparison of changes in BCAA and AAA concentrations by intervention group yielded controversial results that do not confirm or refute our original hypothesis. An analysis of specific body changes, i.e., in fat mass and fat-free mass yielded more conclusive results.

Our analysis of metabolic marker changes according to weight changes post intervention showed that both adolescents who gained weight and those who had lost weight had a decrease in BCAA and AAA concentrations while for HOMA-IR2 index, only those who lost weight had a decrease in their index. Adolescents with obesity or overweight at baseline experienced a greater decrease of their metabolic markers compared to those with a normal weight status. However, it should be noted that adolescents with obesity at baseline had higher BCAA and AAA concentrations at baseline. Therefore, body changes in these adolescents might have induced more substantial changes in BCAA and AAA metabolic pathways compared to overweight or normal weight adolescents. Indeed, there is experimental evidence of a metabolic pathway between BCAA and adipose tissue by Herman et al. who demonstrated by using mice that there was an overexpression of the glucose transporter protein type-4 (GLUT4) which coordinates down-regulation of BCAA metabolizing enzymes in adipose tissue, thus explaining the corresponding decrease in circulating BCAA levels [19].

These results on weight change effects on metabolic markers partially agree with those of Reinehr et al. who observed a decrease of glutamine, methionine and lysophosphatidylcholines levels in adolescents who experienced weight loss, but no change in adolescents who maintained weight after a 1-year lifestyle intervention [20]. A review of short-term obesity-related interventions also pointed out that both diet and diet/physical activity interventions resulted in a weight loss and a metabolic profile improvement in adolescents and children [21]. However, Reinehr et al. raised the lack of direct measure of FM as a limitation in interventional studies [12,20]. When comparing our results from weight change analysis and specific body composition (FM and FFM), the findings underscore these limitations.

A total of 77 adolescents lost weight after the 5-month RASA intervention, while 93 lost FM. When metabolic changes were analyzed according to FM and FFM changes, we noted that BCAA concentrations and HOMA-IR2 significantly decreased among participants who lost FM, but not among those who gained FM, in whom HOMA-IR2 increased. Moreover, among the four body composition groups, the FM^lost/^FFM^gain^ group experienced the greatest decrease of BCAA/AAA concentrations. This suggests that FM loss associated with an FFM gain has a greater impact on related metabolic pathways. As our study is the first to evaluate effects of a weight loss intervention on these metabolic markers using concomitant analysis of body changes, it is difficult to compare results with the existing literature. However, previous studies showed that people with higher muscle mass had better lipid and protein metabolism, which might explain the lower levels of amino acids [22,23]. In addition, a study evaluating the impact of physical activity on IR in women with obesity revealed that changes in skeletal muscles are strongly implicated in IR pathways [24]. Skeletal muscle metabolism and oxygen consumption are favored by a narrow mitochondrial network on the surface of the myocyte membrane or myofibrillar septum [25,26]. If the amount or function of these mitochondria is decreased, skeletal muscle fatty acids are not catabolized and accumulate into diacylglycerol, long-chain acyl-coenzyme A and ceramide, which may lead to IR [25]. This might explain why we observed that FM loss combined with FFM gain is associated with an IR improvement and reduced metabolic marker concentrations.

According to our results, BCAA and AAA changes were more consistent and noticeable compared to HOMA-IR2 after a 5-month intervention, which might indicate that they are more sensitive as metabolic markers. On this matter, Tabák et al. pointed out that changes in traditional biomarkers of T2D (insulin-resistance, fasting, and 2 h post-load plasma glucose) predict early stage only 2 years before its diagnosis, but BCAA and AAA changes were noted 12 years before diagnosis [27]. Therefore, BCAA and AAA are viewed as metabolic markers that can help predict the severity of obesity and its related complications at a much earlier stage than traditional indicators [16].

While this study generated strong findings, it also has some limitations. Of the total group of adolescents, 6.6% were excluded from our study. Since this exclusion was not due to the intervention, there is no apparent selection bias. One concern about this study is the representativeness of the population. The RASA intervention focused only a local college, therefore adolescents in other colleges may have different metabolic characteristics from those in this study. Furthermore, Polynesians are a population with a specific genetics profile and environmental factors that might limit generalization to other populations. Another notable limitation is the fact that we have no information about the diet of adolescents outside the school and no details about other physical activities performed every week. Added to the lack of a control group, these two elements make it hard to attribute the observed results solely to the intervention. Additionally, even if we controlled for gender and age, the most cited confounding factors in the literature, puberty stage, is also an important confounder that should be accounted for when studying the metabolism of adolescents. Hormonal changes in adolescents can have a strong influence on the outcomes of such interventions. Another methodological limitation to consider is the measure of body composition by bioimpedance. While the method is based on the relative stability of hydration in lean, the electrical conduction of body water depends on the amount of electrolytes, which in turn varies with age. This variability could lead to errors in the measure of body composition, especially in children and young adults. However, other tools for measuring body composition such as dual-energy X-ray absorptiometry (DXA) or magnetic resonance imaging (MRI) can be expensive, time-consuming, and not widely available for field studies. Studies have been conducted to determine the variability of results with different measurement tools and several authors have reported a strong correlation between body composition estimated by bioimpedance and that estimated by DXA [28].

Another issue is the length of the intervention. The 5-month RASA intervention could be viewed as short since it is recommended to carry out intervention programs for about 9 to 12 months in order to obtain relevant metabolic results [29]. Finally, the presence of several non-significant changes and overlapping of confidence intervals point out that our post hoc analysis lacks statistical power for stratified analysis. Despite these limits, this study is novel as no interventional study in adolescents had measured BCAA and AAA metabolite changes in relation to concomitant changes in FM and FFM changes. This study also provides information on a unique pediatric population that may be at high risk of developing T2D due to a very high prevalence of overweight. It also provides relatively new markers for assessing metabolic changes following obesity intervention in children and adolescents.

## 4. Materials and Methods

### 4.1. Ethical Statement

This paper is part of a large study called “Ressourses Alimentaires et Santé aux Australes”. The RASA Intervention Research Program was approved by the Ethics Committee of French Polynesia (CEPF/N° 46 on 5 November 2009) and the Ethics Committee of the Centre de Recherche du Centre Hospitalier Universitaire de Québec (CRCHUQ).

### 4.2. Study Design and Participants

The RASA study follows a pre- and post-test design and uses data from a school-based intervention in French Polynesia [18]. This study was conducted from February to June 2011 among adolescents aged 10 to 18 years attending college (equivalent of secondary school) at Mataura (Tubuai Island, Austral Archipelago, French Polynesia). RASA’s main objective was to prevent weight gain among adolescents during the intervention and therefore, to slow down the obesity growth in the school. In total, 242 adolescents attended the college at the time of the study. Medical reasons or refusal to participate were the only exclusion criteria. Parental/guardian consent was obtained for minor children.

Two of the 242 students were excluded from the intervention; one who refused to participate and one for medical reasons. At baseline, the RASA group included 240 students divided into three subgroups according to their college attendance status which determines their level of exposure to the intervention. Externs (n = 14) were those who never ate at the school canteen and slept at home; half-residents (n = 155) were those who ate at the school canteen (lunch only, every day from Monday to Friday) and slept at home, whereas residents (n = 71) were those who ate at the school canteen (3 meals/day, every day) and slept at school (except during holidays). Of the 240 collegians, 8 (3.33%) left college during the school year and were then considered as lost to follow-up. We excluded 6 (2.50%) adolescents from the analysis for lack of complete data on metabolic markers. Finally, 226 adolescents were retained for our analysis. For the analysis, adolescents were divided into two groups reflecting their adherence to the intervention. Those who were living in the school residence thus having their three meals at the canteen (the residents) were considered the most exposed to the intervention (n = 69). Externs and half-residents were considered as partially exposed to the intervention. Due to the small sample size, externs were grouped with the half-residents (n = 157).

### 4.3. Intervention

The 5-month RASA intervention had two components: A school nutrition program and a physical activity facility. For the nutrition component, five cycle menus were elaborated considering dietary rules tailored to teenagers’ needs and the availability of fruits, vegetables, and fish on the island. To achieve that, farmers and fishermen were brought together to determine the seasonal availability of food and were offered an employment contract. In addition, canteen staff received recommendations for food preparation including the reduction of salt, sugar, and fat consumption in foods. As the students were free to eat whatever they wanted during breaks and meals eaten at home, especially those not staying in the school residence, meaning the half-residents and the externs, dietary guidelines and community sensitization were added to the study protocol. To achieve that, each participant received healthy lifestyle documentation before the study started, and this documentation was reviewed during class each month. In addition, food vendors around the college were also provided with education regarding the promotion of fruit, water, and diet drinks. Since residents received weekly food packages from their parents, parents were recommended to ration their quantities and to limit foodstuffs rich in fat and sugar. Finally, information sessions were scheduled before, during, and after the program, to advise parents about the benefits of healthy lifestyles.

In order to increase the energy expenditure of participating students, the weekly amount of physical activity was increased with the addition of 2 to 4 h of training in Polynesian canoes, known locally as Va’a. The choice of the Va’a activity was based on considerable attractiveness of this sport among young Polynesians, as identified by their answers to the 2007 health study. Thus, six Va’a were built for the study. For that component, Mataura College physical education teachers and volunteers supervised the training sessions.

### 4.4. Outcomes

#### 4.4.1. Plasma Amino Acids

Fasting blood samples were collected through cubical vein puncture in collection tubes at the college by the in-house nurses. Blood samples were kept at 4 °C and processed within 3 h of sample collection. Samples were centrifuged at 2000× *g* during 5 min, the plasma transferred in plastic tubes and stored at −20 °C during the first two weeks, and subsequently at −80 °C until analysis. Plasma concentrations of BCAA and AAA (mg/L) were quantified by liquid chromatography coupled with quadrupole time-of-flight mass spectrometry at the Centre de toxicologie du Québec of the Institut National de Santé Publique du Québec (INSPQ) following the method of Roy and colleagues [30]. Concentrations of valine, leucine, and isoleucine were summed to yield the BCAA concentration. AAA concentration was the sum of tyrosine and phenylalanine concentrations.

#### 4.4.2. Homeostasis Model Assessment of Insulin Resistance

HOMA-IR2 index was obtained through the “HOMA calculator” interface developed by the Diabetes Clinical Trials Unit at Oxford University (https://www.dtu.ox.ac.uk/homacalculator/, accessed on 19 January 2017) [31]. Glucose (mmol/L) was measured by a standard enzymatic method and insulin (pmol/L) was measured using double antibody radioimmunoassay method. These measurements were carried out in the laboratory of biochemistry at the CHU de Québec. HOMA-IR2 tertiles were defined according to the distribution of the variable at baseline.

#### 4.4.3. Body Weight and Body Composition

Body weight and body composition (FM and FFM in kilograms) were assessed with a bioelectrical impedance analyzer (Tanita TBF-300). Height was measured barefoot on a hard surface with a freestanding stadiometer and recorded to the nearest centimeter. To ascertain overweight/obesity status, we used BMI values specific for children’s age and gender adopted by the IOTF, and developed on behalf of the International Association for the Study of Obesity [32]. FM refers to the body’s total adipose tissue and FFM to muscle, bone, connective tissue, water, and other non-adipocyte tissues. Four ordinal categories of body composition changes were created: FM gain with FFM loss (FM^gain^/FFM^loss^); gain of FM and FFM (FM^gain^/FFM^gain^); loss of FM and FFM (FM^loss^/FFM^loss^), and FM loss with FFM gain (FM^loss^/FFM^gain^). We hypothesized that the least metabolically advantageous body composition change was FM^gain^/FFM^loss^ and the most advantageous one was FM^loss^/FFM^gain^.

#### 4.4.4. Triglyceride and HDL-Cholesterol

Triglyceride and HDL-cholesterol concentrations were measured in participants’ plasma using the collected blood samples. Analysis of total cholesterol and TG was performed using an Auto-Analyzer II (Technicon Instruments Corporation, Tarrytown, New York). The HDL-cholesterol fraction was obtained after precipitation of the other lipoproteins. Concentrations of both variables are expressed in mmol/L.

### 4.5. Statistical Analysis

The normality of the continuous variables was checked using graphical plots and the Shapiro–Wilk test. Adjusted mean differences of BCAA and AAA concentrations after 5 months were obtained by least squared mean estimates based on generalized estimating equations using SAS PROC MIXED (Minitab’s General Linear Model). At first, residents were compared to externs/half-residents according to weight status and HOMA-IR2 tertiles at baseline. Analyses were adjusted for age, sex, and concentration at baseline of the variable of interest. Co-variance analysis for repeated measurements (rANCOVA) was used to compare the two groups and evaluate group and time effects. Secondly, we compared adjusted mean differences of BCAA, AAA, and HOMA-IR2 index according to body changes. Tests for linear trend across the four categories were assessed using the SAS software PROC GLM CONTRAST (orthogonal polynomial contrast generated). The models were adjusted for age, sex, initial weight status, and value at baseline of the variable of interest. A *p*-value of 0.05 (two-sided) was considered to establish statistical significance. Statistical analyses were performed by SAS software, version 9.4 (SAS Institute, Cary, NC, USA).

## 5. Conclusions

After a 5-month school-based intervention, FM loss coupled to FFM gain induced a greater and significant decrease in metabolic marker concentrations among adolescents. This demonstrates for the first time the importance of considering both changes in FM and FFM occurring in interventional studies. Moreover, BCAA and AAA seem to be sensitive markers of metabolic changes that can make a difference in the evaluation of interventions as well as in the detection of chronic diseases, such as type 2 diabetes.

Nevertheless, larger studies that consider confounding factors such as puberty stage are needed to confirm our findings. Moreover, research to date suggests that adding exercise programs to dietary restriction may promote more favorable changes in body composition than diet alone. Our results demonstrate the relevance of such interventions combining diet and physical activity in the field of adolescent and childhood obesity. Finally, the study design of the RASA intervention also provides an opportunity for a real-world intervention within a practical setting for a developing region such as French Polynesia.

## Figures and Tables

**Table 1:** Baseline characteristics according to attendance status of adolescents in school-based RASA intervention, Austral Islands, French Polynesia.

Characteristics Mean (SD)	TOTAL (n = 226)	Attendance Status		*p*-Value ^†^
		Residents (n = 69)	Externs/ Half-Residents (n = 157)	
Age, years	13.5 (1.6)	14.3 (1.5)	13.2 (1.6)	<0.001
(min., max.)	(10, 18)	(11, 18)	(10, 17)	
Girls, n (%) *	108 (47.79)	37 (53.62)	71 (45.22)	0.24
Weight, kg	69.6 (19.1)	72.7 (17.1)	68.2 (19.9)	0.10
Height, cm	167 (9.46)	168 (8.96)	16 (9.67)	0.15
BMI, kg/m^2^	24.7 (5.5)	25.5 (4.7)	24.4 (5.8)	0.18
Fat-free mass, kg	51.7 (12.4)	53.4 (12.1)	50.9 (12.5)	0.17
Fat mass, kg	17.7 (11.2)	18.6 (10.3)	17.3 (11.6)	0.42
Fat mass, %	24.9 (10.2)	24.6 (10.5)	23.7 (10.1)	0.52
Fasting insulin, pmol/L	108 (92.8)	92.4 (49.1)	115 (112)	0.11
Fasting glucose, pmol/L	4.90 (0.38)	4.76 (0.33)	4.95 (0.40)	0.001
TG, mmol/L	0.96 (0.62)	0.87 (0.82)	1.00 (0.54)	0.09
HDL, mmol/L	1.23 (0.27)	1.26 (0.23)	1.23 (0.29)	0.44
TG/HDL	0.87 (0.66)	0.74 (0.48)	0.93 (0.76)	0.06
Weight status, N (%) *^‡^				0.51
*Normal*	93 (41.15)	25 (36.23)	68 (43.31)	
*Overweight*	56 (24.78)	17 (24.64)	39 (24.84)	
*Obese*	77 (34.07)	27 (39.13)	50 (31.85)	

Abbreviations: ANOVA, analysis of variance; BMI, Body mass index; HDL, high density lipoprotein; IOTF, International Obesity Task Force; SD, Standard deviation; RASA, Ressources Alimentaires et Santé aux Australes; TG, triglycerides. * These variables are not expressed in mean (SD). These are counts with proportions (%). ^†^ The Chi-square test analyzed categorical variables and ANOVA continuous variables (*p* < 0.05). ^‡^ According to IOTF classification.

**Table 2:** Concentrations of BCAA and AAA (Mean (SD)) (mg/L) at baseline and after a 5-month weight reduction intervention in adolescents enrolled in the RASA study, according to weight and attendance status.

				Analysis ^§^		
IOTF Weight and Attendance Status	Baseline ^†^	Post-Intervention ^†^	Change ^‡^ Mean (95% CI)	Group Effect	Time Effect	Group × Time Interaction
					*p* Value	
**All (n = 226)** ^¶^						
**BCAA**				0.95	<0.001	0.48
Residents	59.3 (10.8)	56.5 (9.2)	−3.25 (−6.43 to −0.07)			
Half-Residents/Ext.	61.6 (10.7)	57.3 (8.9)	−4.53 (−6.39 to −2.68)			
**AAA**				0.71	<0.001	0.08
Residents	26.1 (4.7)	25.1 (3.1)	−1.16 (−2.16 to −0.15)			
Half-Residents/Ext.	27.7 (4.6)	25.3 (3.1)	−2.44 (−3.27 to −1.61)			
**Normal weight (n = 93)**						
**BCAA**				0.57	0.11	0.84
Residents	59.8 (11.7)	56.2 (7.4)	−2.68 (−8.04 to 2.67)			
Half-Residents/Ext.	57.5 (9.5)	54.6 (9.2)	−2.09 (−4.92 to 0.74)			
**AAA**				0.30	0.59	0.35
Residents	25.0 (3.2)	25.1 (2.6)	0.24 (−1.30 to 1.78)			
Half-Residents/Ext.	25.6 (4.5)	24.4 (3.1)	−0.87 (−2.15 to 0.40)			
**Overweight (n = 56)**						
**BCAA**				0.52	<0.001	0.26
Residents	64.9 (11.9)	57.9 (7.5)	−8.87 (−13.2 to −4.52)			
Half-Residents/Ext.	61.4 (9.5)	57.0 (9.3)	−5.73 (−8.71 to −2.75)			
**AAA**				0.28	<0.001	0.73
Residents	26.9 (3.2)	24.2 (2.6)	−3.08 (−4.46 to −1.69)			
Half-Residents/Ext.	27.2 (4.6)	25.1 (3.1)	−2.68 (−4.04 to −1.32)			
**Obese (n = 77)**						
**BCAA**				0.62	0.03	0.04
Residents	58.5 (11.8)	57.2 (7.4)	−0.25 (−5.70 to 5.20)			
Half-Residents/Ext.	65.2 (9.6)	59.8 (9.4)	−6.92 (−10.5 to −3.38)			
**AAA**				0.96	<0.001	0.01
Residents	27.2 (3.2)	25.8 (2.6)	−1.24 (−3.02 to 0.54)			
Half-Residents/Ext.	30.6 (4.6)	26.8 (3.5)	−4.39 (−5.83 to −2.95)			

Abbreviations: AAA, aromatic amino acids; BCAA, branched-chain amino acids, IOTF, International Obesity Task Force; SD, Standard deviation; rANCOVA, co-variance analysis for repeated measurements. ^†^ Unadjusted. ^‡^ Adjusted for baseline score of the metabolite concentrations tested, sex, and age. ^§^ The group and time effect was analyzed by rANCOVA. ^¶^ Adjusted for sex, age, and IOTF weight status.

## Data Availability

The data presented in this study are available on request from the corresponding author. The data are not publicly available due to their containing of information that could compromise the privacy of research participants.

## Supplementary Materials

The following supporting information can be downloaded at: https://www.mdpi.com/article/10.3390/metabo12070589/s1. Table S1: Metabolite concentrations at baseline and 5-month post-intervention according to HOMA-IR2 tertiles and attendance status, Table S2: Adjusted mean differences (95% CI) of BCAA and AAA concentrations and HOMA-IR2 according to weight change, Table S3: Adjusted mean differences (95% CI) of plasma BCAA and AAA concentrations and HOMA-IR2 according to body composition change categories.
