# Supplementary material for: Branched-Chain and Aromatic Amino Acids in Relation to Fat Mass and Fat-Free Mass Changes among Adolescents: A School-Based Intervention
Source: Metabolites. 2022 Jun 24;12(7):589. doi: 10.3390/metabo12070589 (PMC9316312; doi:10.3390/metabo12070589)
Supplement: Supplementary file 1 [file metabolites-12-00589-s001.zip › TABLE S1.pdf]

Table S1. Metabolite concentrations (mg/L) at baseline and 5-month post-intervention according HOMA-IR2 tertiles and attendance status

HOMA-IR2 tertiles and attendance status	Baseline[a]	5-month [a]	5-month change [b] Mean (95% CI)	Analysis[c]		
				Group effect	Time	Group x time interaction
					P value	
T1 (n=73)						
**BCAA**				*0.33*	*<.001*	*0.23*
Residents	64.7 (12.2)	57.5 (8.4)	-9.51 (-14.2 to -4.88)			
Half-Residents/Ext.	56.9 (9.7)	53.7 (10.6)	-6.01 (-9.31 to -2.70)			
**AAA**				*0.08*	*<.001*	*0.36*
Residents	25.3 (3.3)	24.5 (2.8)	-1.50 (-2.74 to -0.26)			
Half-Residents/Ext.	25.5 (4.9)	24.7 (3.6)	-2.45 (-3.71 to -1.18)			
T2 (n=86)						
**BCAA**				*0.78*	*0.09*	*0.18*
Residents	55.0 (11.2)	55.9 (7.6)	-0.44 (-4.69 to 3.80)			
Half-Residents/Ext.	60.5 (9.5)	56.(9.3)	-4.19 (-7.34 to -1.03)			
**AAA**				*0.24*	*0.003*	*0.54*
Residents	25.4 (3.1)	24.5 (2.5)	-1.53 (-3.24 to 0.18)			
Half-Residents/Ext.	27.6 (4.8)	25.2 (3.2)	-2.30 (-3.81 to -0.79)			
T3 (n=67)						
**BCAA**				*0.78*	*0.78*	*0.11*
Residents	60.5 (13.2)	58.7 (8.9)	2.55 (-5.20 to 10.3)			
Half-Residents/Ext.	64.6 (9.5)	60.2 (9.6)	-3.60 (-6.74 to -0.46)			
**AAA**				*0.45*	*0.15*	*0.08*
Residents	28.5 (3.6)	27.2 (2.9)	0.25 (-2.16 to 2.66)			
Half-Residents/Ext.	29.1 (4.8)	25.9 (3.3)	-2.59 (-4.05 to -1.13)			

Abbreviation: AAA, aromatic amino acids; BCAA, branched-chain amino acids, HOMA-IR2, homeostasis model assessment of insulin resistance; rANCOVA,co-variance analysis for repeated measurements.

[a]Unadjusted.
[b]Adjusted for baseline score of the metabolite concentrations tested, sex, and age. [c]The group and time effect was analyzed by rANCOVA
