# Supplementary material for: Branched-Chain and Aromatic Amino Acids in Relation to Fat Mass and Fat-Free Mass Changes among Adolescents: A School-Based Intervention
Source: Metabolites. 2022 Jun 24;12(7):589. doi: 10.3390/metabo12070589 (PMC9316312; doi:10.3390/metabo12070589)
Supplement: Supplementary file 1 [file metabolites-12-00589-s001.zip › TABLE S2.pdf]

Table S2. Adjusted [a] mean differences (95% CI) of BCAA and AAA concentrations and HOMA-IR2 according to weight change

	Δ WEIGHT		
	**WEIGHTLOSS**<br>Change ≤ 0kg<br>(n=77)	**WEIGHTGAIN**<br>Change > 0kg<br>(n=149)	**P value[b]**
**Metabolites concentrations**			
BCAA, mg/L	-4.68 (-7.55 to -1.81)	-3.86 (-5.81 to -1.91)	*0.64*
AAA, mg/L	-2.40 (-3.55 to -1.24)	-1.87 (-2.67 to -1.07)	*0.46*
HOMA-IR2	-0.36 (-0.59 to -0.13)	0.15 (0.02 to 0.27)	*<.001*
Fasting insulin, pmol/L	-20.5 (-35.8 to -5.17)	21.1 (4.97 to 37.2)	*<.001*
Fasting glycemia, mmol/L	-0.32 (-0.42 to -0.22)	-0.13 (-0.19 to -0.06)	*0.002*

Abbreviation: AAA, aromatic amino acids; BCAA, branched-chain amino acids, HOMA-IR2, homeostasis model assessment of insulin resistance; IOTF, International Obesity Task Force.

[a]Adjusted for baseline score of the metabolite concentrations tested, sex, and age, intervention group and IOTF weight status

[b] Tests for comparison of means differences between two weight change categories were assessed using the SAS software PROC GLM CONTRAST (orthogonal polynomial contrast generated with estimate statement).
