# Supplementary material for: Branched-Chain and Aromatic Amino Acids in Relation to Fat Mass and Fat-Free Mass Changes among Adolescents: A School-Based Intervention
Source: Metabolites. 2022 Jun 24;12(7):589. doi: 10.3390/metabo12070589 (PMC9316312; doi:10.3390/metabo12070589)
Supplement: Supplementary file 1 [file metabolites-12-00589-s001.zip › TABLE S3.pdf]

Table S3: Adjusted [a] mean differences (95% CI) of plasma BCAA and AAA concentrations and HOMA-IR2 according to body composition change categories

	+ FM			- FM			
**FFM change**	**-FFM** (n=54)	**+ FFM** (n=79)	**TOTAL (n=133)**	**- FFM** (n=33)	**+ FFM** (n=60)	**TOTAL (n=93)**	***P* trend [b]**
**Metabolites**							
BCAA; mg/L	-0.86 (-3.68 to 1.97)	-2.26 (-5.12 to 0.59)	**-1.69 (-3.74 to 0.36)**	-4.95 (-9.04 to -0.87)	-9.10 (-12.1 to -6.15)	**-7.64 (-10.1 to -5.20)**	***<.001***
AAA; mg/L	-1.13 (-2.36 to 0.10)	-1.48 (-2.65 to -0.31)	**-1.33 (-2.19 to -0.48)**	-2.51 (-4.40 to -0.63)	-3.38 (-4.50 to -2.25)	**-3.07 (-2.08 to -4.06)**	***0.007***
HOMA-IR2	0.24 (0.05 to 0.43)	0.23 (0.06 to 0.40)	**0.23 (0.11 to 0.36)**	-0.41 (-0.78 to -0.04)	-0.40 (-0.63 to -0.16)	**-0.40 (-0.60 to -0.20)**	***<.001***
Fasting insulin; pmol/L	17.8 (2.11 to 33.5)	34.5 (7.00 to 61.9)	**27.7 (10.1 to 45.2)**	-20.5 (-45.2 to 4.2)	-23.9 (-39.3 to -8.62)	**-22.8 (-35.9 to -9.51)**	***<.001***
Fasting glycemia; mmol/L	-0.25 (-0.13 to -0.35)	-0.11 (-0.20 to -0.01)	**-0.16 (-0.24 to -0.09)**	-0.33 (-0.45 to -0.21)	-0.17 (-0.30 to -0.05)	**-0.23 (-0.32 to -0.14)**	***0.96***

Abbreviation: AAA, aromatic amino acids; BCAA, branched-chain amino acids, FM, fat mass; FFM, fat free mass; HOMA-IR2, homeostasis model assessment of insulin resistance; IOTF, International Obesity Task Force.

[a] Adjusted for baseline score of the metabolite concentrations tested, sex, and age and IOTF weight status

[b] Tests for linear trend across body composition changes categories were assessed using the SAS software PROC GLM CONTRAST (orthogonal polynomial contrast generated with Proc IML for unequally spaced treatments).
